# Comprehensive Analysis of DNA Methyltransferases Expression in Primary and Relapsed Ovarian Carcinoma

**DOI:** 10.3390/cancers15204950

**Published:** 2023-10-11

**Authors:** Efthymia Papakonstantinou, Ioanna Pappa, Georgios Androutsopoulos, Georgios Adonakis, Ioannis Maroulis, Vasiliki Tzelepi

**Affiliations:** 1 Department of Obstetrics and Gynecology, School of Medicine, University of Patras, 26504 Patras, Greece; med5141@upnet.gr (E.P.); adonakis@upatras.gr (G.A.); 2 Multidimensional Data Analysis and Knowledge Management Laboratory, Computer Engineering and Informatics Department, School of Engineering, University of Patras, 26504 Patras, Greece; bio3536@upnet.gr; 3 Gynecological Oncology Unit, Department of Obstetrics and Gynecology, Medical School, University of Patras, 26504 Patras, Greece; androutsopoulos@upatras.gr; 4 Department of General Surgery, School of Medicine, University of Patras, 26504 Patras, Greece; mario@upatras.gr; 5 Department of Pathology, School of Medicine, University of Patras, 26504 Patras, Greece

**Keywords:** DNA methylation, DNA methyltransferases, DNMT, ovarian cancer, high-grade ovarian cancer, relapsed ovarian cancer

## Abstract

**Simple Summary:**

It is widely accepted that DNA methylation, mediated by DNA methyltransferases, is altered in epithelial ovarian cancer (EOC) and contributes to carcinogenesis, the advancement of the disease and even its chemotherapy resistance. After an immunohistochemical study of a patient cohort from our department and TNM plot, GEPIA2, Kaplan–Meier Plotter, TCGA and Proteomic data analysis for mRNA and protein expression of DNMTs (DNMTA, DNMT2, DNMT3A, DNMT3B and DNMT3L), it is obvious that DNMTs expression is altered in ovarian cancer tissues. DNMT1, DNMT3A, DNMT3B and DNMT3L are overexpressed in primary high-grade EOC. DNMT3A is overexpressed in the early stages only, whereas DNMT1 and DNMT3L are also overexpressed in tumor relapses. On the other hand, DNMT2 is downregulated in high-grade ovarian carcinomas. This can be a pivotal first step in biomarker and/or more effective targeted treatment development for patients with ovarian cancer.

**Abstract:**

Background: Despite recent advances in epithelial ovarian carcinoma (EOC) treatment, its recurrence and mortality rates have not improved significantly. DNA hypermethylation has generally been associated with an ominous prognosis and chemotherapy resistance, but the role of DNA methyltransferases (DNMTs) in EOC remains to be investigated. Methods: In the current study, we systematically retrieved gene expression data from patients with EOC and studied the immunohistochemical expression of DNMTs in 108 primary and 26 relapsed tumors. Results: Our results showed that the DNMT1, DNMT3A, DNMT3B and DNMT3L RNA levels were higher and the DNMT2 level was lower in tumors compared to non-neoplastic tissue, and DNMT3A and DNMT2 expression decreased from Stage-II to Stage-IV carcinomas. The proteomic data also suggested that the DNMT1 and DNMT3A levels were increased in the tumors. Similarly, the DNMT1, DNMT3A and DNMT3L protein levels were overexpressed and DNMT2 expression was reduced in high-grade carcinomas compared to non-neoplastic tissue and low-grade tumors. Moreover, DNMT1 and DNMT3L were increased in relapsed tumors compared to their primaries. The DNMT3A, DNMT1 and DNMT3B mRNA levels were correlated with overall survival. Conclusions: Our study demonstrates that DNMT1 and DNMT3L are upregulated in primary high-grade EOC and further increase in relapses, whereas DNMT3A is upregulated only in the earlier stages of cancer progression. DNMT2 downregulation highlights the presumed tumor-suppressor activity of this gene in ovarian carcinoma.

## 1. Introduction

Ovarian cancer (OC) is the eighth most common cancer and the fifth most common cause of cancer-related death in women [1], representing the deadliest of all gynecological malignances [1]. Mostly occurring in postmenopausal women, the mean age of diagnosis is 65 years [2]. Major risk factors for OC are Hereditary Breast and Ovarian Cancer (HBOC) Syndrome, Lynch Syndrome, hormonal therapy, endometriosis, IVF treatment, use of fertility drugs, late menopause (after the age of 55), early menarche (before the age of 12) and null parity [3]. In 60% of cases, OC is diagnosed at an advanced stage [4]. Due to its asymptomatic nature and the lack of screening methods, it is known as the “whispering” or “silent” cancer [5].

Epithelial ovarian cancer (EOC) is the most common histologic type of ovarian cancer, accounting for 90% of cases. There are five major histological subtypes of EOC: high-grade serous ovarian carcinoma (HGSOC), low-grade serous ovarian carcinoma (LGSOC), mucinous ovarian carcinoma (MOC), clear cell ovarian carcinoma (CCOC) and endometrioid ovarian carcinoma (EnOC) [4]. Each EOC subtype is characterized by different gene mutations that deregulate diverse signaling pathways, and this knowledge should be employed for the development of personalized treatment strategies. HGSOC is the most common and the most aggressive form of EOC, responsible for 70–80% of OC-related deaths [6]. Germline *BRCA1* or *BRCA2* mutations are reported in 15–20% of HGSOC patients [7]. Homologous recombination deficiency (HRD), either sporadic or germline, is seen in nearly half of HGSOC cases [8]. HRD is a marker predictive of response to platinum-based therapy and poly (ADP-ribose) polymerase inhibitors (PARPis). TP53 somatic mutations are observed in 90% of HGSOC cases [9]. Mutations in CCN1, PIK3CA and PTEN tumor suppressor loss are also frequently observed in HGSOCs [10].

LGSOCs are less aggressive tumors with a relatively better prognosis than HGSOCs, occur in younger women (median age of 55 years) and account for less than 5% of all OCs [4]. A small subset of patients has a prior history of a serous borderline tumor (SBT). LGSOCs have a significantly higher expression of ER and PR compared to HGSOCs and are not associated with *BRCA* germline mutations [11], showing *KRAS* and *BRAF* mutations instead [10].

Mucinous, clear cell and endometrioid carcinomas are less frequently seen, with the latter two being associated with endometriosis. Numerous studies have demonstrated an increased incidence of defective mismatch repair (dMMR) and an associated microsatellite instability-high (MSI-H) phenotype in non-serous ovarian cancer (typically tumors of endometrioid or clear cell histology) [12].

Despite the combined use of surgery and chemotherapy in the modern era, mortality rates have not improved significantly over recent decades for OC [13]. LGSOCs are chemoresistant, with a less than 5% response rate to first-line chemotherapy compared to the 80% response rate of HGSOC treated with platinum/taxane drugs [11]. Even though HGSOCs initially respond to platinum/taxane-based therapies, the majority of patients will eventually develop chemotherapy resistance and tumor recurrence [14]. Thus, the detection of novel therapeutic targets that will drive personalized therapy is crucial for improving OC patients’ prognoses [15].

Epigenetic mechanisms, that is, changes in DNA expression without any alteration in DNA sequence, including DNA methylation, histone post-transcriptional modifications and microRNA expression, are considered pivotal in tumor initiation and progression and represent potential therapeutic targets [16]. DNA methylation is a biological process in which the cytosine bases of eukaryotic DNA are converted to 5-methylcytosine. This event usually takes place in CpG dinucleotides located within the promoter region of genes, thereby silencing transcriptional activity [17]. Transcriptional silencing can be inherited to daughter cells following cell division [18] and can be pharmacologically inverted [19]. In cancer cells, the genome is generally hypomethylated [20] compared to normal cells, and this leads to genomic instability and oncogene expression [21,22]. However, hypermethylation is seen in specific promoters, namely those of tumor-suppressor genes, resulting in a reduced expression of the respective protein [23,24].

DNA methylation is mediated by enzymes belonging to the family of DNA methyltransferases (DNMT1, 2, 3A, 3B and 3L). DNMT1 is responsible for maintaining the methylation profile following DNA replication, whereas DNMT3A and DNMT3B mediate de novo methylation [25]. DNMT3A and DNMT3B are also responsible for DNA methylation in germ cell development and embryogenesis. DNMT3L has no catalytic activity but enables DNMT3’s interaction with the histone code (i.e., DNMT3L-DNMT3A heterotetramers). DNMT2 is the fifth member of the family, and, even though it shows structural similarity to the other DNMTs, its DNA methyltransferase activity is rather weak [26]. It is, instead, capable of methylating aspartic acid tRNAs [26]. Thus, DNMT2, also called TRDMT1, represents an RNA methyltransferase.

Alterations in the expression of DNMTs have been shown in various solid tumors and hematologic malignancies and have been associated with diverse clinicopathologic features and survival outcomes [27], frequently independently of other prognostic factors, underscoring their importance as potential drivers in a variety of cancer types. These data rationalized the development of DNA methyltransferase inhibitors (DNMTi), which can restore the expression of tumor suppressor genes [28]. At present, two DNMTi drugs, 5- azacytidine (AZA) and decitabine, have been approved for treating patients with myelodysplastic syndrome (MDS) and acute myeloid leukemia (AML) [29], and are also being tested as therapeutic options in several solid cancers such as colon, ovarian and lung carcinomas [30]. However, many items remain unsolved considering tumor responses, suggesting the presence of alternative ways by which DNMTi may modulate cancer cells [31].

A mounting amount of evidence suggests that DNA methylation is implicated in OC development and progression. Studies have shown that tumor suppressor gene loss in OC is mediated by DNA methylation [32], and many epidemiological studies focus on the role of DNA methylation in OC susceptibility [33]. DNMTs are often overexpressed in various cancer tissues and cell lines, and their levels have been associated with poor survival [34]. However, few studies have analyzed the expression of DNMTs in ovarian carcinomas, with conflicting results, and the prognostic effect of these enzymes in ovarian cancer remains to be investigated [35,36,37].

In the current study, we used publicly available datasets from patients with OC (TNMplot, Gepia2, Protein Data Commons and Kaplan–Meier Plotter) to analyze the expression of DNMTs at the RNA and protein level and correlate it with clinical data. We also analyzed the expression of DNMTs in a cohort of primary epithelial ovarian carcinomas and their relapses using immunohistochemistry in an attempt to provide a thorough understanding of the role of DNMTs in ovarian cancer and identify potentially effective drug targets.

## 2. Materials and Methods

To compare the expression of the DNMTs between normal, tumor and metastatic tissue, we used data from the TNM plot and TCGA for mRNA levels and proteomic data commons for protein levels. To analyze the expression of the markers according to stage, the Gepia 2 platform was used.

### 2.1. TNM Plot

The TNM plot (http://www.kmplot.com, accessed on 5 May 2022) is the largest online transcriptomic database, containing data from nearly 57,000 tissue samples from multiple RNA-seq and microarray datasets. It represents a useful tool for the comparison of gene expression between tumor and normal tissues [38,39]. This database includes 33,520 samples from 3180 gene-chip-based studies from GEO (453 metastatic, 29,376 cancer and 3691 normal samples), 11,010 samples from TCGA (394 metastatic, 9886 cancer and 730 normal samples), 1193 samples from TARGET (1 metastatic, 1180 cancer and 12 normal samples) and 11,215 normal samples from GTEx [38]. Differential expression analyses in a variety of cancer subtypes, primary and metastatic, as well as clinical data are also available, making it possible to identify genes with aberrant expression in a variety of neoplasms.

In the current study, we adopted the TNMplot database to compare the expression of DNMT1, DNMT2, DNMT3A, DNMT3B and DNMT3L in normal, tumor and metastatic ovarian samples. Only datasets utilizing the Affymetrix HGU133, HGU133A_2 and HGU133A platforms were considered because these platforms use identical sequences for the detection of the same gene. The RNA seq data included 133 normal and 374 tumor tissues, and the gene chip data included 46 normal, and 744 primary and 44 metastatic tumor tissues. Statistical significance was computed using Mann–Whitney or Kruskal–Wallis tests.

### 2.2. TCGA

Differential gene expression analyses were implemented, using 319 primary ovarian tumor samples from TCGA (the Genome Cancer Atlas, TCGA-OV project) and 88 normal ovarian samples from GTEx, using the R packages limma and edger [40].

### 2.3. Proteomic Data Commons

The proteomic data commons (PDC) is one of the several repositories within the NCI Cancer Research Data Commons (CRDC) and was developed to advance in-depth analyses of how proteomics are involved in cancer risk, diagnosis, development, progression and treatment. The Cancer Proteogenomic Data Analysis Site (cProSite) (https://cprosite.ccr.cancer.gov/, accessed on 1 August 2023) is a web-based interactive platform that contains data derived from the PDC and currently includes 11 cancer types (breast, colon, head and neck, liver, lung, ovarian, pancreatic, stomach and uterine). Using the cProSite, we compared the quantity and phosphorylation levels of the DNMT1, DNMT3A and DNMT3B proteins between 83 tumors and 19 normal tissues and correlated them with their respective mRNA expression levels.

### 2.4. GEPIA 2

GEPIA 2 (Gene Expression Profiling Interactive Analysis) is a newly developed web server tool. It is used to analyze RNA sequence profiling data from 9736 tumors and 8587 normal samples from the Cancer Genome Atlas (TCGA) and the Genotype-Tissue Expression (GTEx) projects. GEPIA provides a customized analysis of gene expression where users can upload their RNA expression findings and compare them with TCGA and GTEx samples. It is also enhanced with further functions such as differential expression analysis and survival analysis. GEPIA is available at http://gepia.cancer-pku.cn/, accessed on 1 August 2023. We used GEPIA to correlate mRNA DNMTs expression with tumor stage in 404 tumor samples that were included in the TCGA dataset.

### 2.5. Survival Analysis

In order to evaluate the prognostic significance of DNMTs, the Kaplan–Meier plotter and Gepia2 software were used. Overall survival (OS) and progression free survival (PFS) were selected as endpoints. We restricted the analysis to high-grade ovarian serous carcinomas, as these are by far the most common aggressive carcinomas.

The online database Kaplan–Meier plotter (www.kmplot.com, accessed on 1 August 2023) contains gene expression data and survival information from lung, ovarian, gastric and breast cancer patients. To analyze the OS and PFS of 1268 patients with HGSOCs, the patient samples were divided into two groups according to the expression levels of each gene (high vs. low expression). The best-performing threshold was automatically used as a cutoff for determining low vs. high expressions for the Kaplan–Meier plotter. For Gepia 2, we used various thresholds. The data shown were obtained using the thresholds (mostly around 50%) that revealed the most significant results. The log rank *p*-value and hazard ratio (HR) with 95% confidence intervals were calculated and displayed on the webpage. Briefly, the five genes (DNMT1, DNMT2, DNMT3A, DNMT3B and DNMT3L) were uploaded into the database to obtain the Kaplan–Meier survival plots. The log rank *p*-value and hazard ratio (HR) with 95% confidence intervals were calculated and displayed on the webpage. Only the JetSet best probe set was chosen to obtain Kaplan–Meier plots.

### 2.6. Tissue Cohort

#### Patients

We retrieved tissue samples from 108 patients with primary ovarian tumors from the files of the Department of Pathology of the University Hospital of Patras. In 26 of them, tissue from disease relapse was also available. All patients had undergone systematic surgical staging for OEC at the University Hospital of Patras between 2000 and 2017. The mean age of the patients at surgery was 52 years (ranging from 18 to 80 years). Eighty-nine (89) patients had serous tumors (12 borderline tumors, 17 low-grade carcinomas, 60 high-grade carcinomas), and 19 had mucinous tumors (12 borderline tumors and 7 carcinomas). None of the patients had received preoperative systematic therapy. The 26 relapses included 1 SBT, 4 LGSOC, 19 HGSOC, 1 MBT and 1MOC. Relapsed tumors had received adjuvant platinum-based chemotherapy. Table 1 shows the clinicopathologic characteristics of the patients.

All tumors were placed in formalin within 10 min from resection, processed within 48 h and then embedded in paraffin. The hematoxylin and eosin (H&E)-stained slides of the specimens were reviewed by an expert pathologist (VT) in order to determine the histological subtype, grade and tumor (T), lymph node (N), metastasis (M) stage and FIGO stage, the latter two according to the 8th Edition of the AJCC Cancer Staging Manual [41]. A representative formalin fixed-paraffin-embedded (FFPE) tissue block was selected from the tumor for each patient. This study was approved by the Ethical Committee of the University Hospital of Patras (#127/09.03.2021).

### 2.7. Imunohistochemistry

Immunohistochemistry is a rapid, widely available, inexpensive method that gives information about protein expression in a cell- and subcellular-specific manner [42]. In addition, IHC can effectively be used on FFPE samples even with low tumor cell content, and as each cell type is separately evaluated, results in cancer cells are not affected by the presence and abundance of stromal cells [43]. Finally, immunohistochemistry is widely used in everyday practice in pathology departments for tumor biomarker quantification on archival tissue [44]. In order to specifically determine the expression of DNMTs in the epithelium (cancer cells) and, in addition, separately address nuclear and cytoplasmic expression, sections from patient’s tissue blocks were subjected to immunohistochemistry.

Briefly, paraffin-embedded tissue sections, 3 μm thick, were sliced from each tissue block, dried at 60 °C for 15 min, deparaffinized in xylene, rehydrated in a graded alcohol series and washed in tap water. For antigen retrieval, the sections were heated, at 600 W in a microwave for 20 min. Endogenous peroxidase activity was blocked (0.3% H_2_O in methanol) at room temperature for 15 min. The sections were then incubated under the conditions indicated by the manufacturer with primary antibodies against DNMT1, DNMT2, DNMT3A, DNMT3B and DNMT3L. The sources and dilutions of the antibodies used are shown in Table 2. Dako EnVision polymer (Dako EnVision Mini Flex, Dako Omnis, Agilent Technology Inc., Santa Clara, CA, USA, GV823) was used for signal detection. Diaminobenzidine (Dako Omnis, GV823) was used as a chromogen, and Harris hematoxylin was used for nuclear counterstaining. Positive controls for antibody validation were used according to the manufacturer’s instructions. Control experiments without the primary antibody demonstrated that the signals observed were specific.

### 2.8. Evaluation of Immunohistochemical Stains

All slides were observed under light microscopy (BX241, Olympus, Tokyo, Japan). The immunoreactivity was assessed by an expert pathologist (VT) blinded to the pathologic characteristics of each case. Areas with the highest density of positive cells (hot spots) were selected at low-power (×40) magnification. Cell counts were performed at a 400× magnification using a 10 × 10 microscope grid. More than 1000 cells were counted. The intensity and the percentage of positively stained cells were evaluated. The intensity of the staining (1–3) was multiplied by the % of positive tumor cells, and a combined score (H-SCORE) was reported (range 0–300) for each case. The localization (nuclear and cytoplasmic) of a positive signal was also assessed.

### 2.9. Statistical Analysis

In order to summarize patient characteristics and biomarker expression data, various descriptive statistical methods, as well as exploratory analysis techniques, were used. Continuously scaled variables were summarized with descriptive statistical measures, for example, median and standard deviation (SD). The Kolmogorov–Smirnoff/Lilliefors test was used to test whether the data follow a normal distribution. The Kruskal–Wallis test was used in order to perform comparisons between biomarker expressions. The Wilcoxon signed rank test was used for paired comparisons. All reported *p*-values are two-sided at a significance level of 0.05. In order to adjust for multiple comparisons, a Bonferroni correction was used. Analyses were performed using R programming language (R version 4.2.0).

## 3. Results

### 3.1. Aberrant DNMTs Expression Correlates with OΕC Development

**Figure 1:**
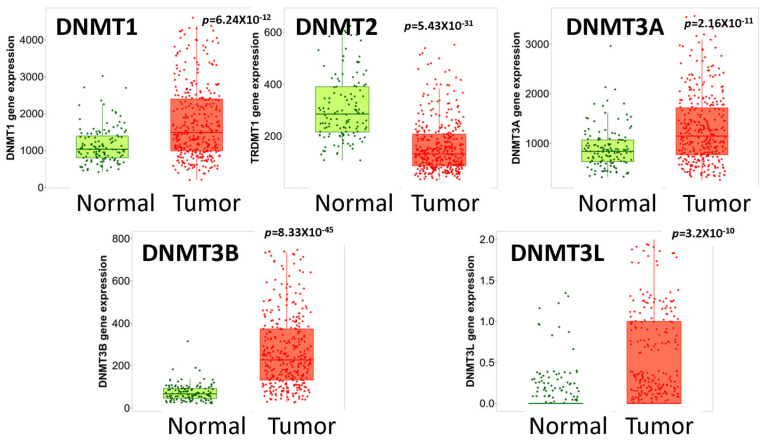
DNMTs mRNA expression in normal vs. tumor tissue (RNAseq data from TCGA, GTEx and TARGET). DNMT1, DNMT3A, DNMT3B and DNMT3L levels are elevated, and DNMT2 levels are decreased in tumor compared to non-neoplastic tissue.

**Figure 2:**
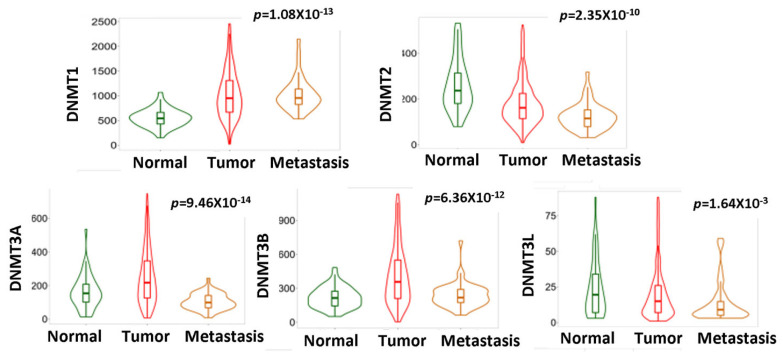
DNMTs mRNA expression in normal vs. neoplastic vs. metastatic tissue (gene chip data from GEO). DNMT3A, DNMT3B, DNMT3L and DNMT2 expression is reduced in metastatic tumors compared to primary neoplasms.

The RNAseq data from The TNMplot (TCGA, GTEx and TARGET projects) revealed that the DNMT1, DNMT3A, DNMT3B and DNMT3L mRNA levels were higher and DNMT2 mRNA expression was lower in HGSOC compared to non-neoplastic tissue (*p* < 0.0001 for all markers) (Figure 1). These results were confirmed with gene chip data from the GEO series (TNMplot) for DNMT1, DNMT2, DNMT3A and DNMT3B (*p* < 0.001 for all markers) but not for DNMT3L (*p* = 0.237) (Figure 2).

Additionally, the gene chip data (Figure 2) from the GEO series (TNMplot) indicated that DNMT3A (*p* < 0.0001), DNMT3B (*p* < 0.0001) and DNMT3L mRNA (*p* < 0.0001) expression was reduced in metastatic tumors compared to the primaries and almost reached the levels of normal tissues, while DNMT1 expression was maintained at high levels in metastasis and was not statistically different from that of the primary tumors. Regarding the expression of DNMT2/TRDMT1, a further decline in metastatic tumors compared to primary carcinomas was noted (*p* < 0.001) (Figure 2). According to the above results, it is clear that DNMT1 expression is maintained at high levels throughout the spectrum of ovarian cancer development and progression.

**Figure 3:**
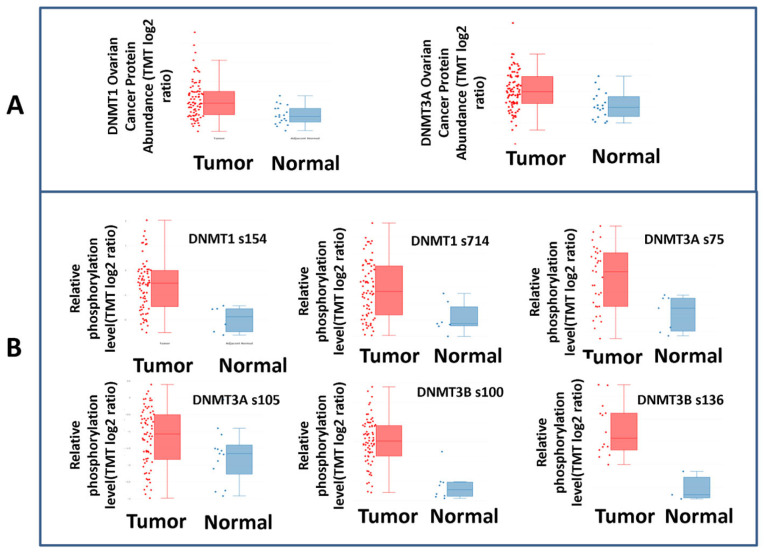
DNMT levels in tumor vs. normal tissue (cProSite analysis). (**A**) DNMT1 and DNMT3A protein expression is elevated in ovarian tumors vs. normal tissues. (**B**) DNMT1, DNMT3A and DNMT3B protein phosphorylation sites in ovarian tumors vs. normal tissues.

In line with the mRNA results, the cProSite analysis showed higher levels of the DNMT1 and DNMT3A proteins in ovarian cancers compared with normal adjacent tissues (*p* < 0.0001 and *p* < 0.0005, respectively), even though the DNMT1 and DNMT3A proteins did not correlate with their mRNA expression levels (Figure 3).

Further strengthening the role of DNMTs in EOC, a cProSite analysis showed that the phosphorylation levels of the DNMT1 protein at s154 and s714, of the DNMT3A protein at s75 and s105 and of the DNMT3B protein at s100 and s136 were significantly increased in the tumors compared to normal tissues (*p* = 0.0004, *p* = 0.0012, *p* = 0.001, *p* = 0.037, *p* < 0.0001 and *p* = 0.0027, respectively) (Figure 3).

### 3.2. DNMTs Expression Is Altered with the Progression of EOC

**Figure 4:**
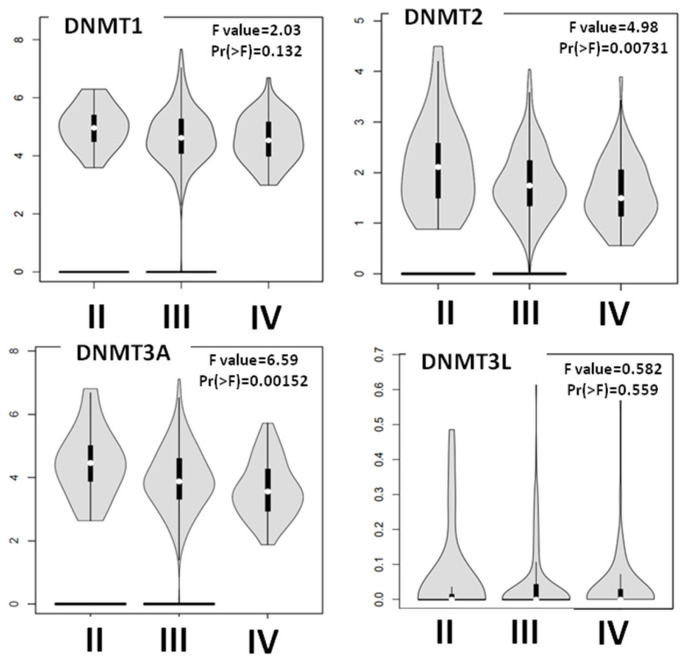
DNMTs mRNA expression according to stage. DNMT2 and DNMT3A levels decreased from Stage II to Stage IV.

Next, we used the Gepia2 and TCGA databases to explore the correlation between individual DNMTs and the stage progression (Figure 4). A Gepia2 analysis revealed that the DNMT3A and DNMT2 levels decreased from Stage II to Stage IV (*p* = 0.00152 and *p* = 0.00731, respectively). In agreement with this result, a statistical analysis of the TCGA data showed a higher expression of DNMT3A in Stage-II tumors compared to Stage-IV ones (*p* = 0.00152). Νo correlation between either DNMT1 or DNMT3L mRNA expression and clinical stage was detected on both platforms. Gepia2 did not include data on the stage-specific expression of DNMT3B.

### 3.3. DNMTs Expression Correlates with Patients’ Prognoses

**Figure 5:**
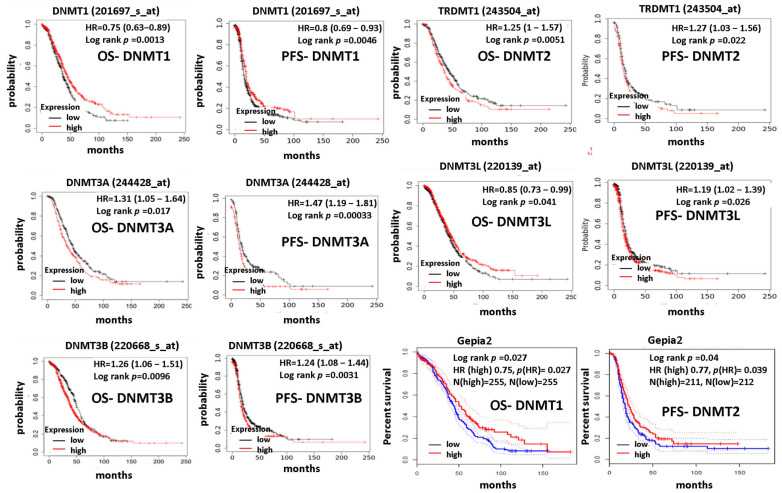
DNMTs mRNA expression and survival in ovarian carcinoma.

The KM plotter curves demonstrated that high mRNA levels of DNMT1 were associated with better OS and PFS survival (*p* = 0.013 and *p* = 0.0046, respectively) (Figure 5). On the contrary, high mRNA levels of DNMT3A and DNMT3B were correlated withworse OS (*p* = 0.017 and *p* = 0.0096, respectively) and PFS (*p* = 0.00033 and *p* = 0.0031, respectively) (Figure 5). DNMT2 mRNA expression was also associated with marginally worse OS and PFS survival (*p* = 0.051 and *p* = 0.022, respectively) (Figure 5). DNMT3L mRNA levels showed ambiguous results as they were associated with better OS (*p* = 0.041) but worse PFS (*p* = 0.026) (Figure 5).

The Gepia 2 survival plots confirmed the association of DNMT1 mRNA overexpression with favorable overall survival (*p* = 0.027) (Figure 5). Furthermore, DNMT2/TRDMT1 was associated with poor DFS (*p* = 0.04) but not OS. Νo statistical correlations were found between the other DNMTs mRNA levels and patients’ prognoses.

### 3.4. Expression of DNMTs in a Cohort of EOC Patients

**Figure 6:**
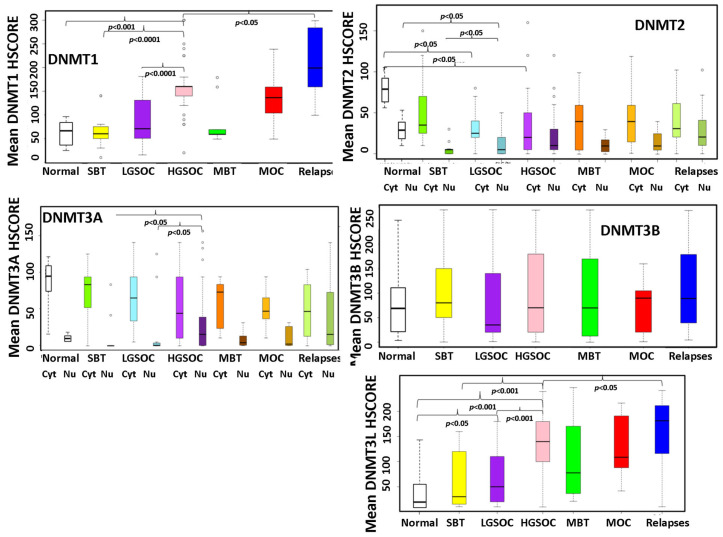
Boxplots of the expression of DNMTs in association with histologic type and relapses (SBT: serous borderline tumor, LGSOC low-grade serous ovarian carcinoma, HGSOC: high-grade serous ovarian carcinoma, MBT: mucinous borderline tumor, MOC: mucinous ovarian carcinoma).

**Figure 7:**
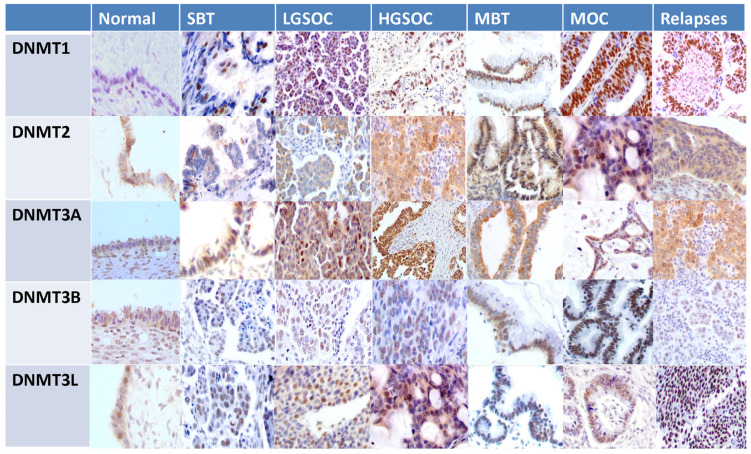
Representative images from DNMT immunohistochemistry (SBT: serous borderline tumor, LGSOC low-grade serous ovarian carcinoma, HGSOC: high-grade serous ovarian carcinoma, MBT: mucinous borderline tumor, MOC: mucinous ovarian carcinoma).

In order to validate the results of the publicly available database analyses, we performed an immunohistochemical evaluation of DNMT protein expression in 108 tissue samples from patients with ovarian serous and mucinous tumors. Both the tumor and the non-neoplastic ovarian surface epithelium were evaluated. DNMT1, DNMT3B and DNMT3L demonstrated nuclear staining, whereas DNMT2 and DNMT3A exhibited nuclear and cytoplasmic staining. Table 3 and Table 4 summarize the mean expression of the markers in primary and relapsed tumors and their correlation with histologic type and FIGO tumor stage. A boxplot graphical illustration of DNMT expression in the various histologic types is shown in Figure 6. Representative images from tissue sections are shown in Figure 7.

The statistical analysis, generally in line with the results of the publicly available datasets, showed that the nuclear expression of DNMT1 was highly elevated in HGSOCs compared to non-neoplastic ovarian epithelial cells, SBTs and LGSOCs (*p* = 0.04 *p* < 0.001 and *p* < 0.001, respectively) (Table 3, Figure 6). Similarly, DNMT2 nuclear expression was reduced in SBT and LGSOC (*p* = 0.03), and cytoplasmic expression was lower in LGSOCs and HGSOCs compared to non-neoplastic tissue (*p* = 0.045) (Table 3, Figure 6 and Figure 7). Nuclear DNMT3A expression was significantly upregulated in HGSOC tumors in comparison with non-neoplastic tissue, SBTs and LGSOCs (*p* = 0.02) (Table 4, Figure 6 and Figure 7). Μοreover, DNMT3L protein expression was increased in HGSOCs and LGSOCs compared with non-neoplastic tissue (*p* < 0.001 and *p* < 0.005, respectively) and in HGSOCs compared with LGSOCs (*p* < 0.001) and SBTs (*p* < 0.001) (Table 4, Figure 6 and Figure 7).

Like serous carcinomas, mucinous carcinomas also displayed a higher expression of DNMT1 and DNMT3L in comparison with non-neoplastic tissues (*p* = 0.042 and *p* = 0.045, respectively) (Table 3 and Table 4, Figure 6 and Figure 7).

Relapses demonstrated significantly elevated DNMT1 and DNMT3L protein expression compared to the corresponding primary HGSOCs (*p* = 0.02 and *p* = 0.032, respectively) (Table 3 and Table 4, Figure 6).

As far as DNMT3B is concerned, no statistically significant alteration was found either between its expression and histological type or between relapses and primary tumors (Table 3). Νo statistical difference was observed in DNMT expression according to FIGO stages (Table 3 and Table 4).

## 4. Discussion

In the present study, we systematically retrieved data regarding the mRNA and protein expression of DNMT1, DNMT2, DNMT3A, DNMT3B and DNMT3L in epithelial ovarian carcinoma samples from interactive web applications such as TNMplot, GEPIA2, Kaplan–Meier Plotter and Proteomic data commons. We performed differential gene expression analyses between the tumor and normal samples and a correlation of mRNA expression levels with FIGO stage. Then, we investigated the correlation of DNMTs’ expressions with patients’ prognoses. Finally, using immunohistochemistry, we evaluated DNMTs’ protein expressions in 108 tissue samples from patients with primary and relapsed ovarian tumors and correlated them with normal tissues and histological subtypes. Ιt should be noted that the expression of the DNMT2 and DNMT3L proteins in ovarian carcinomas by immunohistochemistry has not been reported in the literature before.

Our results showed that DNMT1 expression was elevated from non-neoplastic to tumor tissue at the mRNA and protein levels and further increased with grade progression and in tumor relapses, and its phosphorylation levels were also increased in carcinomas. DNMT3A levels increased from non-neoplastic to tumor tissue at both the mRNA and protein level and further increased with increasing the grade of the tumors, although their levels were lower in higher stages and in metastatic tumors. DNMT3B expression was increased at the mRNA level from non-neoplastic to tumor tissue and decreased in the metastases compared to primary foci, but no difference was noted at the protein level. DNMT3L was increased in the primary tumors compared to non-neoplastic tissue at the mRNA and protein level and was further increased in relapses. In contrast to the other DNMTs, the DNMT2 levels seemed to decrease from non-neoplastic to tumor tissue at the mRNA and protein level and further decrease with stage progression. To the best of our knowledge, this is the first study to address the differential expression of DNMTs according to the histology of OEC and between primary and relapsed tumors at the protein and cellular/subcellular level by immunohistochemistry. This is important as we and others [45,46] have shown that mRNA levels do not always correlate with protein levels, which is attributed to translational regulation, differences in protein in vivo half-lives, and differences in experimental conditions [47]. In addition, using immunohistochemistry, the specific cellular and subcellular expression of the markers was assessed, a feature that PCR and RNA sequencing techniques cannot address [48,49].

Previous studies have shown that DNMT expressions are upregulated in various malignancies (colon, prostate, breast, liver and leukemia) [50,51] and that their expression is associated with aggressive pathologic characteristics [51,52]. The results regarding the expression of DNMTs in ovarian tumors in the literature are equivocal. This is probably a result of the different patient populations studied and the different methodologies used. In addition, as we have shown in this study and reviewed here [53], DNMT expression depends on OEC histologic subtype.

One of the first studies of DNMT expression in ovarian tumors used cancer cell lines and showed an increased expression of DNMT1 and DNMT3B, but not DNMT3A mRNA levels, in ovarian cancer cell lines compared to normal ovarian surface epithelial cells [54]. In patient samples, and similarly to our results, the DNMT3A protein level was found to be elevated in ovarian cancer tissues compared to normal ovary tissues [55]. Importantly, and in agreement with our results, the protein and mRNA levels did not correlate with each other [55]. Another study showed a significantly lower expression of the DNMT3A1 and DNMT2 mRNAs, the latter being in agreement with our results, but a higher expression of the DNMT3B1/B2 mRNA isoforms in carcinomas than in low malignant potential tumors, and that DNMT3B1/3B2 mRNA levels were associated with the methylation status of *CDH13*, *MLH-M2B*, *SEZ6L* and *MINT31-M1B* [37]. In accordance with this, using qPCR in 63 ovarian cancer patient samples, DNMT3B1 and DNMT3B3 overexpression were seen in advanced stages and high-grade serous carcinomas [56]. More recently, the expressions of DNMT1, DNMT3A and DNMT3B were examined by immunohistochemistry in ovarian cancers and benign tumors. The data showed higher DNMT3A and lower DNMT3B protein expression in ovarian cancers compared to that of the benign tumors, whereas DNMT1 expression showed no difference [35]. In contrast, using qRT-PCR and immunohistochemistry, DNMT1 and DNMT3B expressions were shown to be elevated in carcinomas compared to non-neoplastic tissue and further enhanced in higher-stage carcinomas [57].

Taken together and in accordance with our findings, there is abundant evidence in the literature of the aberrant expression of DNMTs in ovarian carcinomas compared to non-neoplastic cells or benign tumors, with further deregulation in high-grade ovarian carcinomas and in metastases/relapses. Considering our comprehensive analysis of DNMT expressions across the progression of OEC, we hypothesize that DNMT1 and DNTM3A seem to play a role in tumor initiation, DNMT3B in tumor progression and DNMT3L in tumor relapse, whereas DNMT2 may have an opposite role, acting as a tumor-suppressor gene. These stage-specific roles of distinct DNMTs account for the progression of other neoplasms as well, such as prostate carcinomas [58]. In addition, others have hypothesized a similar carcinogenesis model in which DNMT3A and DNMT3B are specifically recruited during tumor initiation and promotion, with a subsequent downregulation of their expression, whereas DNMT1 is involved in tumor progression [59]. These findings support the notion that alterations in DNMT expression might contribute to the development and progression of high-grade ovarian carcinomas, i.e., through the establishment of a CpG island methylator phenotype in ovarian cancer.

We also examined the association of DNMT expressions with patients’ survival and showed that DNMT3A, DNMT3B and DNMT2 mRNA levels were correlated with a bad prognosis and DNMT1 expression with a favorable prognosis. The correlation of DNMTs with prognoses is ambiguous and seems to be cancer specific. Some studies have shown that DNMT levels are associated with an ominous prognosis [52], while others have found that the expression of these molecules, in particular DNMT3A, is associated with a good prognosis [27,52]. Regarding ovarian carcinoma, the literature regarding the prognostic role of DNMT expressions is rather sparse. Using immunohistochemistry, Bai et al. showed that the expressions of DNMT1 and DNMT3B were marginally associated with better overall survival [35], the first being in agreement with our results. The combined expression of both markers had a better statistical correlation [35]. An analysis of DNMT3B isoform expression using qPCR in 63 OEC cases showed that DNMT3B1 and DNMT3B3 overexpression were associated with poor prognoses [56]. This agrees with our results, although we did not specifically analyze the different isoforms of the molecule. Similarly, in agreement with our findings, DNMT3A has been found to be associated with a poor prognosis [55]. Taken together, these findings show that DNMT1, DNMT3A and DNMT3B may have a prognostic role in OEC. Further studies with a higher number of patients and a combination of techniques will be needed to validate this hypothesis.

Regarding DNMT1, an interesting finding was that the phosphorylation levels at s154 were increased in tumors compared to normal tissues. In human mammary epithelial cells, it has been shown that the elevated DNMT1 protein levels are not a result of increased mRNA levels but rather an increase in protein half-life due to the deleted destruction domain that is required for its proper ubiquitination and degradation [60]. It has also been suggested that phosphorylation on Ser154 by cyclin-dependent kinases may play a role in controlling DNMT1 activity and protein stability, leading to increased DNMT1 activity [61]. Thus, our findings highlight protein phosphorylation as one of the mechanisms of DNMT1 overexpression in OEC.

DNMT3L is involved in DNMT3A function as it forms a complex with DNMT3A2 and prevents DNMT3A2 from being degraded. Restoring the DNMT3A protein level in DNMT3L-deficient embryonal stem cells (ESC) partially recovers DNA methylation [62]. DNMT3L is expressed in ESC and downregulated in differentiated embryonal cells, thus being associated with pluripotency [63]. The available data regarding DNMT3L mRNA and protein expression in different cancer subtypes are very limited. An overexpressiοn of the DNMT3L protein has been shown in embryonal carcinoma but not in other types of testicular germ cell tumors [64]. It has also been shown to be overexpressed in prostate carcinomas, especially high-grade ones [51]. Its expression in OEC has not been studied before. Ιn our study, we observed an overexpression of the DNMT3L protein in high-grade carcinomas compared to low-grade tumors and non-neoplastic epithelium and in relapses compared to primary neoplasms. Further study of the role of DNMT3L in de novo DNA methylation in ovarian tumors is warranted.

DNMT2 (TRDMT1) is an aspartic acid tRNA methyltransferase, with weak, if any, DNA methyltransferase activity [26]. DNMT2 mutations have been reported in human tumors and are associated with alterations of its methylation activity [65]. DNMT2 upregulation has been reported in GIST [66], glioma [67], prostate carcinoma [51] and clear cell renal cell carcinoma [68]. Its expression in OEC has not been studied before. In the present study, we showed both the cytoplasmic and nuclear expression of DNMT2/TRDMT1, as has been reported previously [26,51]. It is reasonable and expected that a cytosine-5 methyltransferase such as DNMT2, with activity towards both DNA and RNA, would function in both cellular compartments. The database analysis showed that DNMT2 was decreased in carcinomas compared to non-neoplastic tissue. We also noticed that both cytoplasmic and nuclear DNMT2 expression gradually decreased from borderline to high-grade serous tumors, although it did not reach a statistically significant level. It has previously been reported that DNMT2/TRDMT1 is required for efficient homologous recombination and that a loss of its function makes the cells sensitive to PARP inhibitors [69]. This observation is of particular importance in ovarian cancer patients, where homologous recombination deficiency (HRD) and PARP inhibitor therapy are the treatments of choice for at least half of the patients with high-grade serous ovarian tumors. An analysis of DNMT2 expression in ovarian cancer could contribute to the selection of patients as candidates for PARP inhibitor therapy.

Even though an overexpression of DNMTs has not been linked with global DNA methylation status and/or the hypermethylation of specific genes that contribute to carcinogenesis [37], DNMTs have been correlated with tumor progression and patients’ prognoses, and therefore, they may represent potential prognostic biomarkers and therapeutic targets [37,70]. A recent, very promising study showed that the overexpression of DNMT1 and DNMT3B is highly linked to the genome-wide hypermethylation profile in prostate cancer and paved the way for studying their function in many malignances [71]. DNMT inhibitors (DNMTi) have shown promising results in ovarian cancer in preclinical models by enhancing IFNγ-mediated [72] and type-I interferon-mediated inflammatory responses [73]. In addition, DNMTis seem to work synergistically with other therapies. The administration of DNMTis seems to enhance sensitivity to platinum treatment [74], immunotherapy [75] and PARP inhibition [76].

## 5. Conclusions

In conclusion, our study demonstrated an altered expression of DNMTs in epithelial ovarian cancer and a differential expression of individual DNMTs during cancer progression. Namely, we hypothesize that DNMT1 and DNTM3A seem to play a role in tumor initiation, DNMT3B in tumor progression and DNMT3L in tumor relapse, whereas DNMT2 may have an opposite role. We hope that these data might be translated into future therapeutic approaches, as biomarker expression is of outmost importance for developing treatment strategies. Nevertheless, future studies are needed to further validate the role and prognostic significance of DNMT alterations in ovarian cancer.

## Figures and Tables

**Table 1:** Clinicopathologic characteristics of the patients from the OEC cohort.

	SBT Ν = 12	LGSOC Ν = 17	HGSOC Ν = 60	MBT Ν = 12	MOC Ν = 7	Total N = 108
Mean Age	38	63	57	41	51	55
Figo Stage						
1	12	7	13	12	4	48
2		2	4		1	7
3		8	41		2	51
4			2			2
T stage						
T1	12	7	13	12	4	48
T2	0	2	7	0	1	10
T3	0	8	40	0	2	50
N Stage						
N0	12	15	40	12	7	86
N1	0	2	20	0	0	22

SBT: serous borderline tumor, LGSOC: low-grade serous ovarian carcinoma, HGSOC: high-grade serous ovarian carcinoma, MBT: mucinous borderline tumor, MOC: mucinous ovarian carcinoma.

**Table 2:** Conditions used and source of antibodies.

Antibody	Clone	Source	Antigen Retrieval	Dilution- Incubation	Positive Control
DNMT1	Mouse monoclonal H-12	Santa Cruz, CA, USA	1mM EDTA-NaOH pH8	1:50/2 h/room temperature	Lymph node
DNMT2	Rabbit polyclonal	Santa Cruz, CA, USA	10mM citrate buffer pH6	1:150/2 h/room temperature	Placenta
DNMT3a	Mouse monoclonal 64B1446	Abcam, UK	1mM EDTA-NaOH pH8	1:250/1 h/room temperature	Testicle
DNMT3B	Mouse monoclonal 52A1018	Abcam, UK	1mM EDTA-NaOH pH8	1:150/1 h/room temperature	Lymph node
DNMT3L	Rabbit polyclonal	Novus Biologicals, Littleton, CO, USA	1mM EDTA-NaOH pH8	1:150/2 h/room temperature	Testicle

**Table 3:** Mean expression of DNMT1 and DNMT2 in association with histologic type, FIGO Stage and relapses.

		N	DNMT1	*p* Value	DNMT2 Nucleus	*p* Value	DNMT2 Cytoplasm	*p* Value
Non-neoplastic (non)		20	70	vs. SHGOC *p* = 0.04 vs. MOC *p* = 0.04	30		85	
Histologic type	SBT	12	68.3	vs. HGSOC < 0.0001	6.3	vs. non *p* = 0.03	52.9	
	LGSOC	17	89.6	vs. HGSOC < 0.0001	11.9	vs. non *p* = 0.03	32.6	vs. non *p* = 0.045
	HGSOC	60	159.6		21.2		31.3	vs. non *p* = 0.045
	MBT	12	82		10.9		41.1	
	MOC	7	136.8	vs. non *p* = 0.042	15.7		44.4	
FIGO Stage	1	48	100.2		15.2		47.8	
	2	7	132.5		7.5		70	
	3	51	147.5		17.7		28.9	
	4	2	85		5		60	
Relapses		26	210.4	vs. HGSOC *p* = 0.02	28.2		36.2	

SBT: serous borderline tumor, LGSOC low-grade serous ovarian carcinoma, HGSOC: high-grade serous ovarian carcinoma, MBT: mucinous borderline tumor, MOC: mucinous ovarian carcinoma.

**Table 4:** Mean expression of DNMT3A, DNMT3B and DNMT3L in association with histologic type, FIGO Stage and relapses.

		N	DNMT3a Nucleus	*p* Value	DNMT3a Cytoplasm	*p* Value	DNMT3b	*p* Value	DNMT3L	*p* Value
Non- neoplastic (non)		20	10	vs. HGSOC *p* = 0.02	95		70		20	vs. LGSOC < 0.005 vs. HGSOC < 0.001
Histologic type	SBT	12	20.2	vs. HGSOC *p* = 0.02	138.3		103.3		62.5	vs. HGSOC < 0.001
	LGSOC	17	28.06	vs. HGSOC < *p* = 0.02	123.75		71.1		68.1	vs. HGSOC < 0.001
	HGSOC	60	58.3		102.6		95.1		133.1	
	MBT	12	14		109.1		104.1		100.8	
	MOC	7	21.1	vs. non *p* = 0.04	96.2		71.7		127.8	vs. non *p* = 0.045
FIGO Stage	1	48	25.4		113.3		94.9		94.4	
	2	7	37.8		153.3		82.5		55.8	
	3	51	56.6		102.5		90.6		126.5	
	4	2	7.5		70		60		60	
Relapses		26	67		87.8		108.7		157.3	vs. SHGOC *p* = 0.032

SBT: serous borderline tumor, LGSOC low-grade serous ovarian carcinoma, HGSOC: high-grade serous ovarian carcinoma, MBT: mucinous borderline tumor, MOC: mucinous ovarian carcinoma.

## Data Availability

Data will be available upon reasonable requests.
